# A novel prognostic model based on epithelial-mesenchymal transition-related genes predicts patient survival in gastric cancer

**DOI:** 10.1186/s12957-021-02329-9

**Published:** 2021-07-19

**Authors:** Wanting Song, Yi Bai, Jialin Zhu, Fanxin Zeng, Chunmeng Yang, Beibei Hu, Mingjun Sun, Chenyan Li, Shiqiao Peng, Moye Chen, Xuren Sun

**Affiliations:** 1 Department of Gastroenterology, First Affiliated Hospital of China Medical University, Shenyang, Liaoning China; 2 Department of Gastrointestinal Endoscopy, First Affiliated Hospital of China Medical University, Shenyang, Liaoning China; 3 Department of Endocrinology and Metabolism, First Hospital of China Medical University, Shenyang, Liaoning China

**Keywords:** Gastric cancer, Epithelial-mesenchymal transition, Gene, Survival

## Abstract

**Background:**

Gastric cancer (GC) represents a major malignancy and is the third deathliest cancer globally. Several lines of evidence indicate that the epithelial-mesenchymal transition (EMT) has a critical function in the development of gastric cancer. Although plentiful molecular biomarkers have been identified, a precise risk model is still necessary to help doctors determine patient prognosis in GC.

**Methods:**

Gene expression data and clinical information for GC were acquired from The Cancer Genome Atlas (TCGA) database and 200 EMT-related genes (ERGs) from the Molecular Signatures Database (MSigDB). Then, ERGs correlated with patient prognosis in GC were assessed by univariable and multivariable Cox regression analyses. Next, a risk score formula was established for evaluating patient outcome in GC and validated by survival and ROC curves. In addition, Kaplan-Meier curves were generated to assess the associations of the clinicopathological data with prognosis. And a cohort from the Gene Expression Omnibus (GEO) database was used for validation.

**Results:**

Six EMT-related genes, including *CDH6*, *COL5A2*, *ITGAV*, *MATN3*, *PLOD2*, and *POSTN*, were identified. Based on the risk model, GC patients were assigned to the high- and low-risk groups. The results revealed that the model had good performance in predicting patient prognosis in GC.

**Conclusions:**

We constructed a prognosis risk model for GC. Then, we verified the performance of the model, which may help doctors predict patient prognosis.

## Background

Gastric cancer (GC), a worldwide health problem, represents the fifth commonest malignancy and the third deadliest cancer after lung and colorectal cancers [1]. In 2018, there were more than 1,000,000 new cases, and the incidence rate in men was twice that of women [2]. Although studies have shown that chemotherapy, palliative radiotherapy, and other treatments are effective in improving patient survival, GC still causes many deaths [3]. This may result from the atypical early symptoms of GC, causing patients to miss the optical time for treatment and to experience adverse prognosis [4]. Currently, effective methods for accurate prognostic evaluation of GC are still lacking. Therefore, it is essential to construct a model for effectively predicting patient survival in GC.

Epithelial-mesenchymal transition (EMT) represents an important molecular program in diverse cellular events such as wound healing, tissue fibrosis, and cancer progression [5]. Studies have demonstrated that EMT is closely related to tumor heterogeneity, metastasis, and therapeutic resistance in cancer cells [6]. EMT can promote cancer cell invasion into the stroma and is correlated with cancer stem cell traits and increased tumorigenicity [7]. Meanwhile, the EMT/inflammation axis contributes to the metastatic stage and poor outcome of multiple cancer entities and confers chemoresistance to primary tumors [8]. Multiple lines of evidence suggest that many genes, such as *TSP50*, *CD44*, and *FOXA1*, regulate EMT in GC [9–11]. Because EMT is a vital process in GC progression, studying the role of related genes in GC is valuable.

In this study, based on TCGA and MSigDB data, differentially expressed genes associated with EMT were selected. Then, univariable and multivariable Cox regression analyses were carried out for identifying overall survival (OS)-related genes. Next, a risk model was constructed to predict and evaluate patient prognosis in GC. We finally used additional bioinformatic and statistical methods to validate this model.

## Methods

### Data acquisition and processing

The original gene expression data of the TCGA gastric dataset were acquired from the TCGA database (https://portal.gdc.cancer.gov/repository). The RNA sequencing data of GC patients were retrieved. Clinical features, including survival status, survival time, age, gender, grade, stage, T stage, N stage, and M stage, were adopted in the current study. The “HALLMARK_EPITHELIAL_MESENCHYMAL_TRANSITION” gene list containing 200 genes was obtained from MSigDB v7.2(http://www.gsea-msigdb.org/gsea/msigdb/index.jsp). To identify differentially expressed epithelial-mesenchymal transition-related genes (ERGs), we utilized the “limma” package in the R software 4.0.2 to analyze mRNA sequencing data for these 200 ERGs, with significance threshold set at *p*< 0.05.

### Identification of EMT-related gene signature

After merging clinical information and the expression data of ERGs, univariable Cox regression analysis was performed for screening genes related to OS with *p*< 0.05. Then, multivariable Cox proportional regression analysis was performed for the screened genes to identify those independently related to OS.

### Construction of prognosis model

A risk score formula was generated for prognosis based on multivariable Cox regression analysis. Cases were classified into two categories, including the high- and low-risk groups, according to the median patient risk score. Then, survival curves, areas under the receiver operating characteristic (ROC) curves (AUCs) for 1-year, 3-year, and 5-year overall survival were utilized to determine the prediction accuracy of the constructed model.

### Prognostic value validation of EMT-related genes

In order to assess whether the above risk score independently predicts GC prognosis, univariable and multivariable Cox regression analyses were carried out. The expression levels of the six detected ERGs in normal and tumor tissues were examined, and Kaplan-Meier survival analysis based on different clinicopathological parameters was conducted. We also chose a cohort (GSE62254) from the GEO database (https://www.ncbi.nlm.nih.gov/geo/) for validating the prognostic value of the established risk model.

### Assessment of immunocyte infiltration

The Tumor IMmune Estimation Resource (TIMER) database (https://cistrome.shinyapps.io/timer/) represents a comprehensive platform that can analyze immune infiltration systematically in multiple cancers [12]. By exploring the abundance of B cells, CD8+ T cells, CD4+ T cells, macrophages, neutrophils, and dendritic cells in gastric cancer, we could evaluate the possible associations of the hub genes in GC with the six immune cell types.

### Statistical analysis

All of the data analyses were performed using the R software and Perl languages. *P* < 0.05 indicated statistical significance. Cox regression analyses were conducted to screen survival-associated ERGs. The survival curves were plotted by the Kaplan-Meier method to show differences in OS between high/low-risk patients and the log-rank test was utilized to assess the significance of the differences. And the ROC curves and the corresponding AUC values were used to evaluate the predictive ability of the model by using the “survival ROC” package.

## Results

### Identification of differentially-expressed epithelial-mesenchymal transition-related genes

Gene expression data for GC patients and their clinicopathological information were obtained from the TCGA database. Subsequently, 200 ERGs from the “HALLMARK_EPITHELIAL_MESENCHYMAL_TRANSITION” gene set in the MSigDB website were obtained. This database supplies a list of 200 genes that have been shown to be correlated with EMT. Next, we analyzed the retrieved gene expression data and screened for differentially expressed ERGs by using the package “limma” in R (P< 0.05). After screening, we selected 371 gastric cancer patients for analysis. The clinical characteristics of GC patients are shown in Table 1.

**Table 1:** Clinical information of GC patients from TCGA database

Patients characteristics		*n*
Age, years	≤ 65	163
	> 65	205
Gender	Female	133
	Male	238
Grade	G1	10
	G2	134
	G3	218
Stage	Stage I	50
	Stage II	111
	Stage III	149
	Stage IV	38
T stage	T1	18
	T2	78
	T3	167
	T4	100
N stage	N0	108
	N1	97
	N2	74
	N3	74
M stage	M0	328
	M1	25

### Identification of survival associated ERGs in GC

To assess potential associations of ERGs with OS in GC, univariable Cox regression analysis was carried out to identify ERGs that were significantly correlated with survival. A total of 20 ERGs were identified (*P* < 0.05) (Table 2). Then, multivariable Cox analysis was carried out to determine whether the associations of ERGs with OS were independent. The most significant ERGs were included in the model, with the corresponding coefficients.

**Table 2:** Survival associated ERGs screened by univariable analysis in patients with gastric cancer in the TCGA database

Genes	HR	HR.95 L	HR.95H	*P* value
CALU	1.0091	1.0019	1.0163	0.0126
CDH11	1.0431	1.0142	1.0728	0.0032
CDH6	1.2952	1.1122	1.5083	0.0008
COL5A2	1.0052	1.0003	1.0100	0.0357
CTHRC1	1.0103	1.0041	1.0166	0.0010
DAB2	1.0321	1.0102	1.0545	0.0037
FAP	1.0744	1.0137	1.1387	0.0154
FBN2	1.2319	1.0044	1.5110	0.0452
INHBA	1.0203	1.0030	1.0379	0.0211
ITGAV	1.0238	1.0088	1.0391	0.0017
LOX	1.0257	1.0090	1.0427	0.0024
LUM	1.0013	1.0003	1.0023	0.0062
MATN3	1.0652	1.0293	1.1024	0.0003
PDGFRB	1.0080	1.0012	1.0149	0.0208
PLOD2	1.0504	1.0205	1.0811	0.0008
POSTN	1.0047	1.0017	1.0077	0.0018
SERPINE1	1.0018	1.0005	1.0031	0.0049
SPARC	1.0013	1.0004	1.0022	0.0021
VCAN	1.0215	1.0096	1.0336	0.0003
VEGFC	1.0907	1.0191	1.1672	0.0121

Six genes (*CDH6*, *COL5A2*, *ITGAV*, *MATN3*, *PLOD2*, and *POSTN*) were identified, which may serve as important prognosis predictors (Table 3). Totally, 5 ERGs were correlated with poorer prognosis (HR > 1), and their overexpression may reduce survival. The remaining 1 ERG (*COL5A2*) was correlated with better prognosis (0 < HR< 1), and its overexpression may be beneficial to survival.

**Table 3:** Identification of the six ERGs involved in the risk model by multivariable analysis in GC patients

Genes	Coef.	HR	HR.95 L	HR.95H	*P* value
CDH6	0.2159	1.2410	1.0400	1.4809	0.0166
COL5A2	-0.0116	0.9884	0.9785	0.9984	0.0239
ITGAV	0.0208	1.0210	1.0025	1.0398	0.0252
MATN3	0.0488	1.0500	1.0111	1.0903	0.0111
PLOD2	0.0379	1.0386	1.0010	1.0776	0.0437
POSTN	0.0052	1.0052	1.0008	1.0096	0.0193

### Construction of the ERGs-related prognosis risk model

**Fig. 1:**
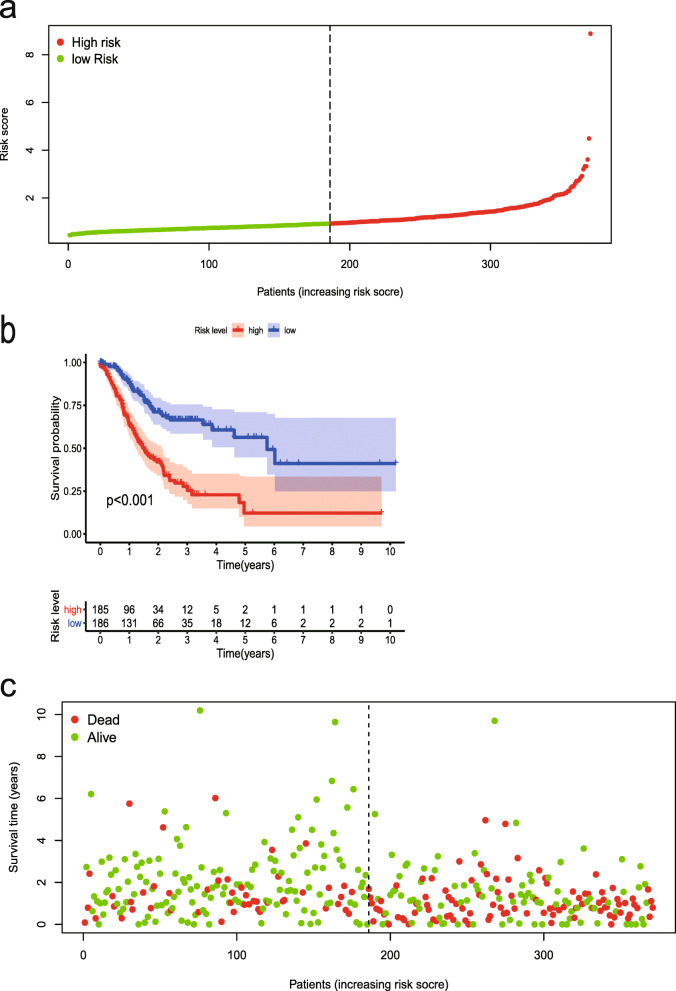
The risk score was established with six ERGs to predict overall survival in GC. **a** Risk scores of GC patients with different risk levels. **b** Survival analysis of the risk model. **c** Patients’ distribution of survival status

**Fig. 2:**
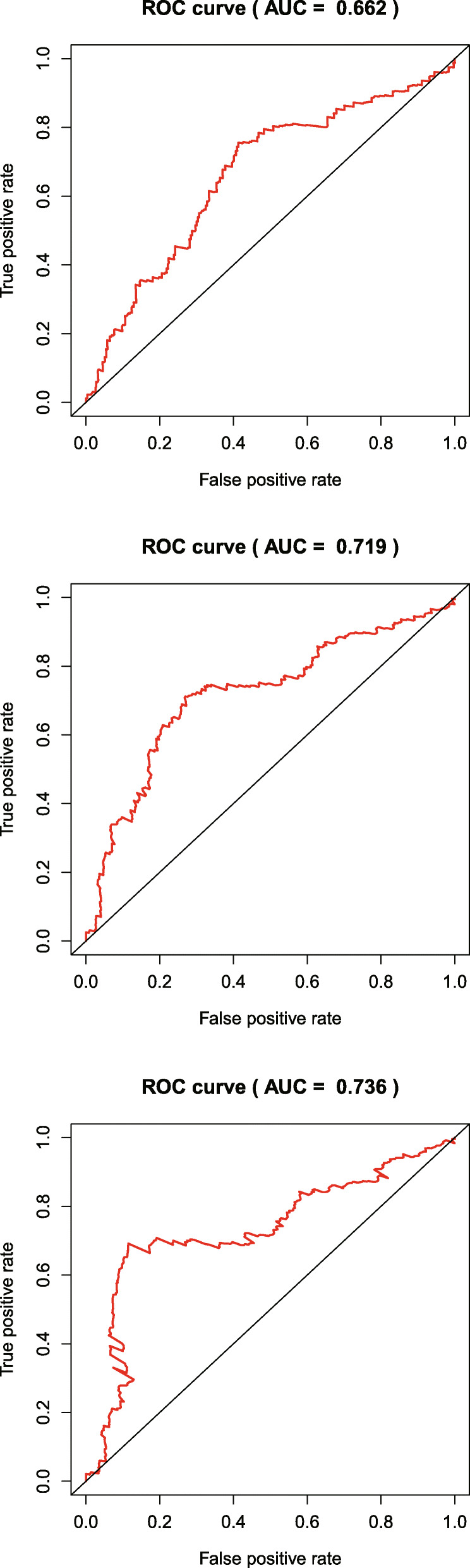
The ROC curves for determining 1-, 3-, and 5-year survival

Based on gene expression data and regression coefficients, a prognostic model was ultimately established. The prognostic risk score was calculated according to the following formula: risk score = 0.2159 × *CDH6* expression − 0.0116 × *COL5A2* expression + 0.0208 × *ITGAV* expression + 0.0488 × *MATN3* expression + 0.0379 × *PLOD2* expression + 0.0052 × *POSTN* expression. According to individual risk scores, all patients were categorized into the low- and high-risk groups as described above (Fig. 1a). In survival analysis, individuals with a low-risk score had a higher survival rate compared with patients with a high-risk score (*p* < 0.001) (Fig. 1b). This suggested that the model was accurate for predicting the prognosis of low- and high-risk gastric cancer patients. Then, GC patients were ranked by risk score to assess the associations of survival status with survival time (Fig. 1c). The scatterplot depicts the survival statuses of patients with different risk scores, and the mortality rate of patients increased with the risk score. Then, the 1-, 3-, and 5-year ROC curves were generated to verify the ability of the risk model to predict prognostic; the obtained AUCs were 0.662, 0.719, and 0.736, respectively, indicating an acceptable predictive performance (Fig. 2).

### Age and risk score are independent prognostic predictors of GC

**Fig. 3:**
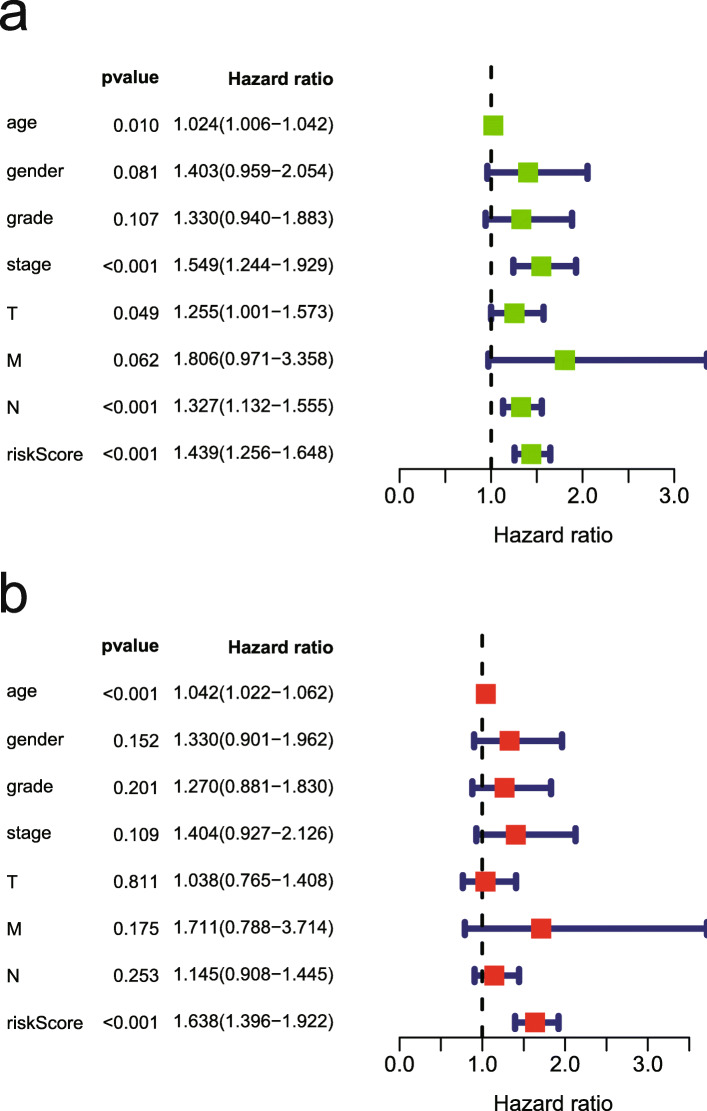
Analysis of each clinicopathological parameter in GC patients in the TCGA database. **a** Univariable Cox regression analysis. **b** Multivariable Cox regression analysis

Univariable and multivariable analyses were performed to examine the prognostic strengths of the risk score as well as select clinicopathological parameters. Univariable Cox analysis revealed that age, stage (T or N), and risk score were OS-associated indicators (Fig. 3a). Subsequently, in multivariable Cox analysis, age and risk score were independent predictors of prognosis (*p* < 0.05) (Fig. 3b).

### Expression levels and validation of six ERGs in GC

**Fig. 4:**
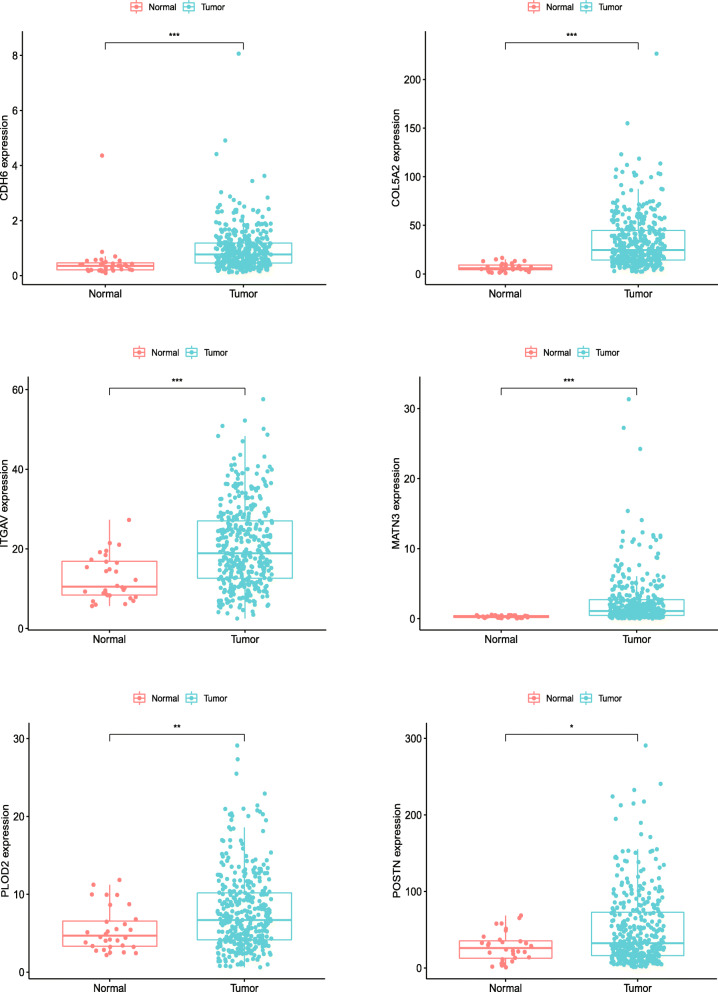
Expression of the six ERGs in gastric cancer tissue samples and noncancerous tissue specimens. *P < 0.05, **P < 0.01, ***P < 0.001

**Fig. 5:**
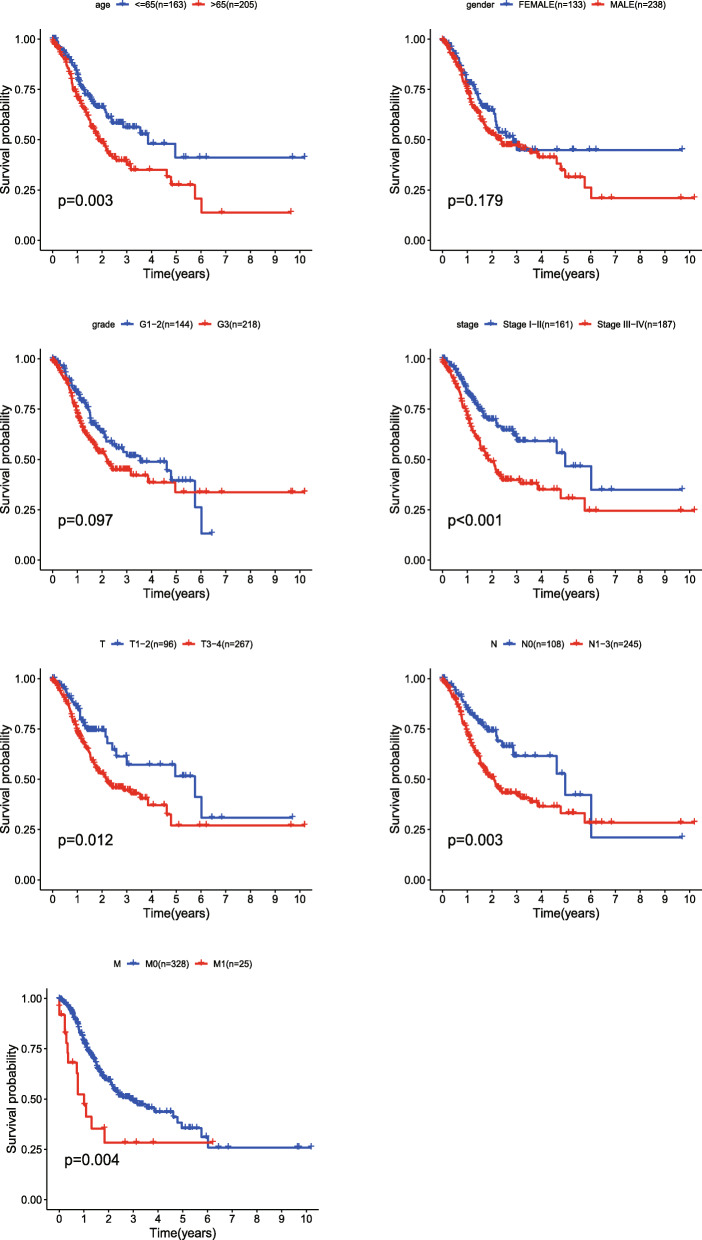
Kaplan-Meier survival analysis of different clinicopathological parameters that correlated with patient survival

**Fig. 6:**
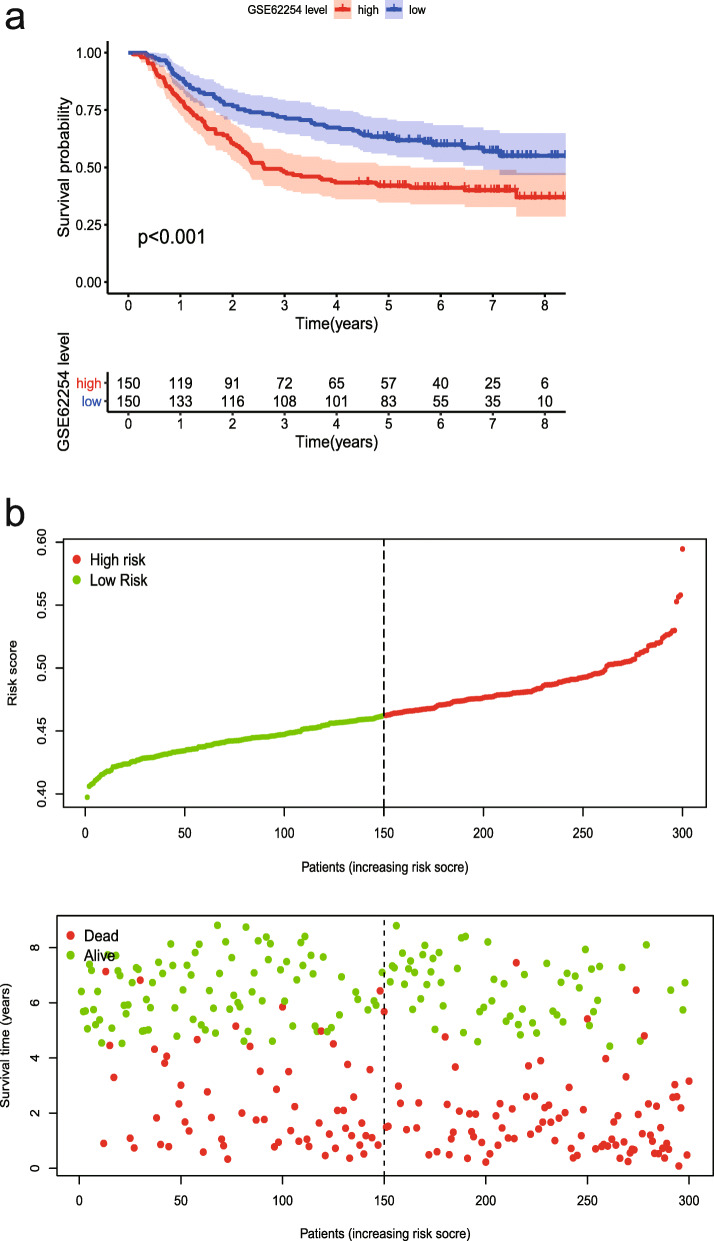
Analysis of GC patients in GSE62254 cohort. **a** Kaplan-Meier survival analysis in GSE62254 cohort. **b** Risk score assessment in GSE62254 cohort

**Fig. 7:**
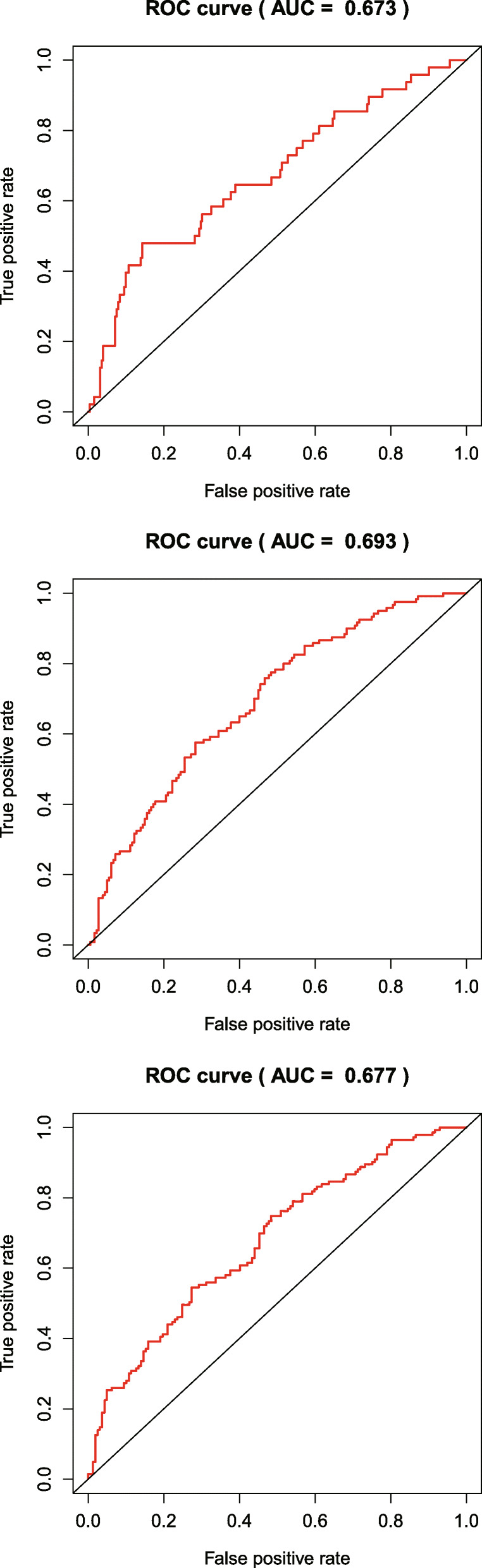
Time-dependent ROC curves of the GSE62254 at 1, 3, and 5 years

**Fig. 8:**
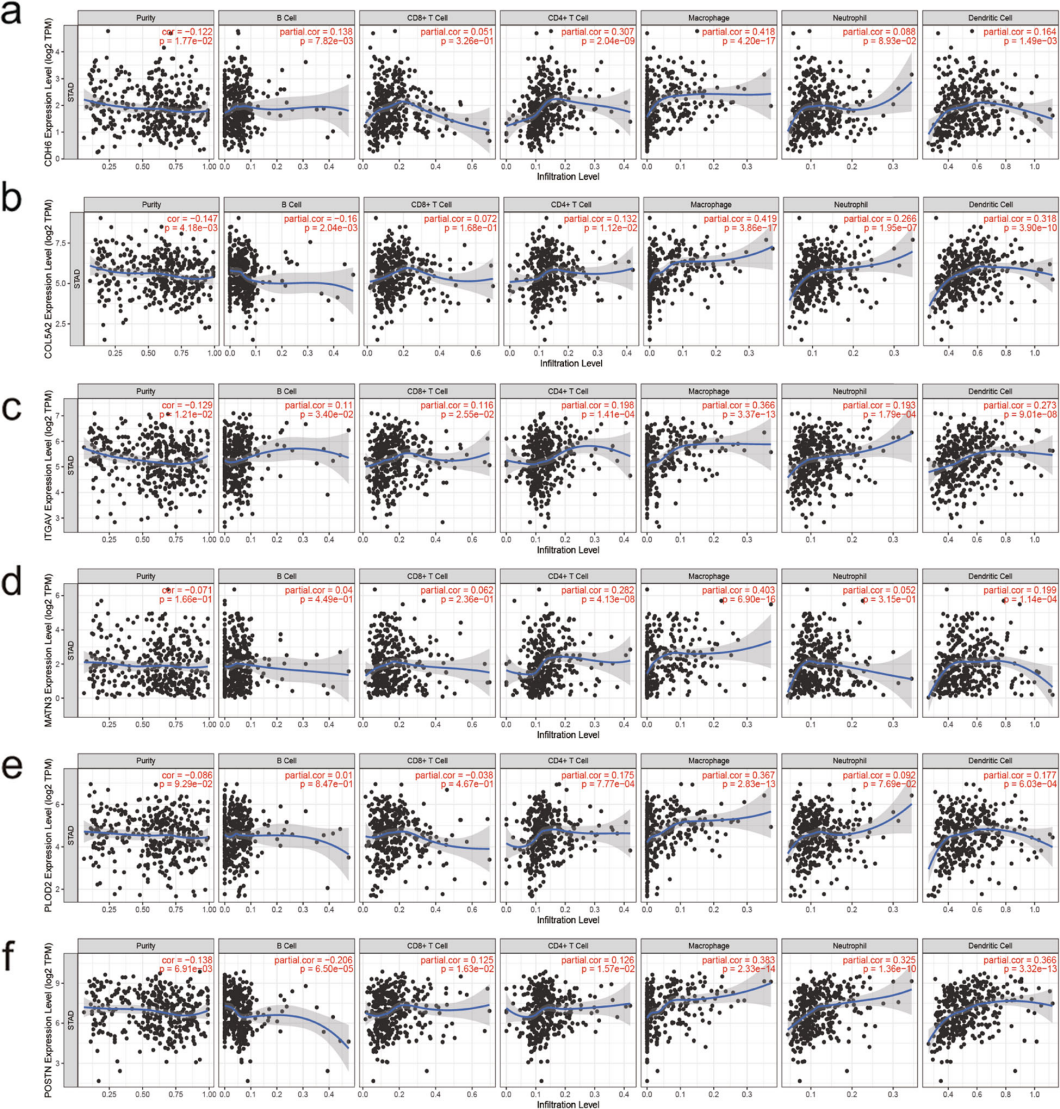
Associations of the expression of the six ERGs with immune infiltration levels. **a** *CDH6*. **b** *COL5A2*. **c** *ITGAV*. **d** *MATN3*. **e** *PLOD2*. **f** *POSTN*

The expression levels of the six genes in GC and normal tissue specimens were next compared. These six ERGs all showed upregulation in GC specimens versus normal gastric tissue samples (*P* < 0.05) (Fig. 4). In addition, clinicopathological parameters were collected from the TCGA database. Kaplan-Meier curve analysis revealed that age, stage, T stage, N stage, and M stage had significant associations with GC patient survival (Fig. 5). Then, we applied the new model to 300 GC samples from GSE62254 to validate our findings. The patients were also divided into two categories by median risk score, and the KM survival curve showed significant results (*P* < 0.001) (Fig. 6). And the AUC values of the ROC curves (0.673 at 1 year, 0.693 at 3 years, 0.677 at 5 years) indicated that this risk model had a satisfactory prognostic value (Fig. 7). Furthermore, the TIMER website was utilized to evaluate the associations of the six ERGs with tumor purity and immune cell infiltration in gastric cancer (Fig. 8). The results showed that *MATN3* and *PLOD2* were not associated with tumor purity, whereas all six genes were significantly associated with CD4+ T cell, macrophage, and dendritic cell levels.

## Discussion

Gastric cancer remains a globally important pathology, with elevated mortality. GC represents a major social burden and peritoneal metastasis is common in advanced GC cases [13]. Postoperative prognosis varies according to the types of GC, and thus, improvements in GC’s treatment are urgently required [14]. In EMT, epithelial cells forfeit their apical-basal polarity and cell-cell adhesion features, becoming invasive cells with mesenchymal characteristics [15]. The relationship between gastric cancer and EMT has been widely reported, with many studies assessing the related genes. For instance, *REC8* inhibits EMT in GC cells by controlling *EGR1* expression and binding to it [16]. In addition, tumor-associated neutrophils contribute to GC cell migration and invasion through IL-17a-induced EMT [17]. Alpha B-crystallin (*CRYAB*) also promotes GC cell migration and invasion through EMT under the control of NF-κB signaling [18]. Although EMT-related genes have been assessed for their functions and mechanisms, the link between ERGs and prognosis in GC remains unclear.

To date, increasing amounts of attention focus on the construction of cancer models. The prognosis of cancer can be predicted by screening out genes related to biological processes, such as the effect of glycolysis-related genes on the prognosis of hepatocellular carcinoma and the association of autophagy-related genes with the survival of lung cancer [19, 20]. However, there is not much discussion about the relationship between EMT-related genes and gastric cancer. Hence, considering the importance of EMT in gastric cancer, it is reasonable to speculate that EMT-associated genes could be applied in GC prognosis. Here, expression data for 371 GC samples and the respective clinical data were retrieved from the TCGA database, as well as a list of 200 ERGs from the MSigDB. Univariable and multivariable Cox regression analyses were performed to obtain six genes associated with survival in GC. According to the established formula, risk scores for individual patients were calculated, based on which cases were significantly stratified. Survival analysis and ROC curves were applied to assess the predictive value of this risk model. As shown above, the risk score and age were determined as independent OS-associated factors. Furthermore, the expression levels of the six genes were higher in tumor tissue samples compared with noncancerous specimens. We also found that the developed prognostic model was correlated with clinicopathological data. And the analysis of the samples in GSE62254 confirmed the predictive ability of the model. The associations of genes with immune cell infiltration were also discussed. We found that the six ERGs are closely related to immune cells, and they are all positively correlated with the degree of CD4+ T cell, macrophage, and Dendritic Cell infiltration in GC. As for B cells, the expression of CDH6 and ITGAV was positively correlated with it, but the expression of COL5A2 and POSTN was negatively correlated with it. Furthermore, the expression of ITGAV and POSTN was positively associated with the degree of CD8+ T cell and neutrophil infiltration, and there was a positive correlation between COL5A2 expression and neutrophil. These findings jointly suggest that the six EMT-related genes play critical roles in GC prognosis, and the new model has a high prognostic predictive value.

Previous studies have reported the roles of these genes in GC. *COL5A2* is related to prognosis in patients with GC [21]. In bladder cancer, cases with low *COL5A2* amounts show improved clinicopathological phenotypes [22]. *COL5A2* is also involved in the carcinogenesis of colorectal cancer [23]. *ITGAV*, belonging to the integrin family of extracellular matrix receptors, is implicated in multiple cancers. Downregulation of *ITGAV* can cause the inhibition of GC cell proliferation, migration, and invasion, indicating a critical role for *ITGAV* in GC progression [24]. *MATN3*, a protein-coding gene, might affect GC occurrence and development, acting as an oncogene [25]. In addition, *MATN3* is differentially expressed and aberrantly methylated in GC [26]. *PLOD2* is an important regulator of peritoneal dissemination in GC patients, leading to poor prognosis [27]. *POSTN* affects cancer cell proliferation via ERK and may promote EMT [28]. *POSTN* overexpression is a known risk factor for GC, inducing metastasis and aggravating malignant behavior [29]. *CDH6*, a class II Cadherin, induces EMT during embryonic development and shows abnormal reactivation in malignant tumors [30]. Different from other mesenchymal biomarkers that are broadly found in cancer, *CDH6* may preferentially participate in the late stage of EMT, with direct association with cell invasiveness [31]. However, *CDH6*’s function in GC remains undefined. Therefore, further research for discovering the function of *CDH6* is urgently required. Besides, there are other studies on gastric cancer. Follistatin-like 1 (FSTL1) is a kind of glycoprotein, its high expression is associated with poor prognosis and has a positive correlation with immune infiltration in GC patients [32]. CTLA-4 polymorphisms have a tight relationship with digestive system malignancies including gastric cancer, it related to predisposition to GC [33]. RNAs are also involved in the development of gastric cancer. For example, miR-489 overexpression can inhibit the survival, invasion, and migration of gastric cancer cells, and circulating lncRNAs play an important role in the early diagnosis of GC [34, 35]. Furthermore, the clinical results of gastric cancer can be predicted by constructing nomogram [36].

So far, most studies have assessed the relationship between a single gene and prognosis in GC. Here, we constructed a dependable prognostic risk model for GC based on sequencing data for ERGs and clinicopathological features from the TCGA database. This might provide a reliable method for predicting patient survival in GC. However, some limitations are worth noting. First, we established the risk model by using the TCGA database, and further validation of this model should be carried out in vitro, in vivo, and in clinical trials. Secondly, the immune statuses of these six genes deserve further investigation.

## Conclusions

This study has developed a six-gene prognostic risk model based on EMT, which could provide a reference for assessing whether gastric cancer patients are at high risk. This risk model could help better manage patients and predict prognosis. These findings may provide novel insights into the relationship between GC and EMT.

## Data Availability

The datasets analyzed during the current study are available in the TCGA database (https://portal.gdc.cancer.gov/repository), the MSigDB website (http://www.gsea-msigdb.org/gsea/msigdb/index.jsp), and the GEO database (https://www.ncbi.nlm.nih.gov/geo/).

## Abbreviations

GC: Gastric cancer
EMT: Epithelial-mesenchymal transition
TCGA: The Cancer Genome Atlas
GEO: Gene Expression Omnibus
ERGs: EMT-related genes
MSigDB: Molecular Signatures Database
OS: Overall survival
ROC: Receiver operating characteristic
TIMER: The Tumor IMmune Estimation Resource

## References

[1] Thrift AP, El-Serag HB (2020). Burden of gastric cancer. Clin Gastroenterol Hepatol..

[2] Bray F, Ferlay J, Soerjomataram I, Siegel RL, Torre LA, Jemal A (2018). Global cancer statistics 2018: GLOBOCAN estimates of incidence and mortality worldwide for 36 cancers in 185 countries. CA Cancer J Clin..

[3] Sitarz R, Skierucha M, Mielko J, Offerhaus GJA, Maciejewski R, Polkowski WP (2018). Gastric cancer: epidemiology, prevention, classification, and treatment. Cancer Manag Res..

[4] Wang Y, Chen S, Tian W, Zhang Q, Jiang C, Qian L, Liu Y (2019). High-expression HBO1 predicts poor prognosis in gastric cancer. Am J Clin Pathol..

[5] Nieto MA, Huang RY, Jackson RA, Thiery JP (2016). Emt: 2016. Cell..

[6] Zhang Y, Weinberg RA (2018). Epithelial-to-mesenchymal transition in cancer: complexity and opportunities. Front Med..

[7] Diepenbruck M, Christofori G (2016). Epithelial-mesenchymal transition (EMT) and metastasis: yes, no, maybe?. Curr Opin Cell Biol..

[8] Suarez-Carmona M, Lesage J, Cataldo D, Gilles C (2017). EMT and inflammation: inseparable actors of cancer progression. Mol Oncol..

[9] Cao QH, Liu F, Li CZ, Liu N, Shu M, Lin Y, Ding L, Xue L (2018). Testes-specific protease 50 (TSP50) promotes invasion and metastasis by inducing EMT in gastric cancer. BMC Cancer..

[10] Xiao WS, Li DF, Tang YP, Chen YZ, Deng WB, Chen J, Zhou WW, Liao AJ (2018). Inhibition of epithelial-mesenchymal transition in gastric cancer cells by miR-711-mediated downregulation of CD44 expression. Oncol Rep..

[11] Lin M, Pan J, Chen Q, Xu Z, Lin X, Shi C (2018). Overexpression of FOXA1 inhibits cell proliferation and EMT of human gastric cancer AGS cells. Gene.

[12] Li T, Fan J, Wang B, Traugh N, Chen Q, Liu JS, Li B, Liu XS (2017). TIMER: A web server for comprehensive analysis of tumor-infiltrating immune cells. Cancer Res..

[13] Wang Z, Chen JQ, Liu JL, Tian L (2019). Issues on peritoneal metastasis of gastric cancer: an update. World J Surg Oncol..

[14] Xue J, Yang H, Huang S, Zhou T, Zhang X, Zu G (2021). Comparison of the overall survival of proximal and distal gastric cancer after gastrectomy: a systematic review and meta-analysis. World J Surg Oncol..

[15] Du B, Shim JS. Targeting epithelial-mesenchymal transition (EMT) to overcome drug resistance in cancer. Molecules. 2016;21(7):965. 10.3390/molecules21070965 PMC6273543 27455225

[16] Zhao J, Geng L, Duan G, Xu W, Cheng Y, Huang Z (2018). REC8 inhibits EMT by downregulating EGR1 in gastric cancer cells. Oncol Rep.

[17] Li S, Cong X, Gao H, Lan X, Li Z, Wang W, Song S, Wang Y, Li C, Zhang H, Xue Y, Zhao Y (2019). Tumor-associated neutrophils induce EMT by IL-17a to promote migration and invasion in gastric cancer cells. J Exp Clin Cancer Res..

[18] Chen D, Cao G, Qiao C, Liu G, Zhou H, Liu Q (2018). Alpha B-crystallin promotes the invasion and metastasis of gastric cancer via NF-kappaB-induced epithelial-mesenchymal transition. J Cell Mol Med..

[19] Jiang L, Zhao L, Bi J, Guan Q, Qi A, Wei Q, He M, Wei M, Zhao L (2019). Glycolysis gene expression profilings screen for prognostic risk signature of hepatocellular carcinoma. Aging (Albany NY)..

[20] Wang X, Yao S, Xiao Z, Gong J, Liu Z, Han B, Zhang Z (2020). Development and validation of a survival model for lung adenocarcinoma based on autophagy-associated genes. J Transl Med..

[21] Shen H, Wang L, Chen Q, Xu J, Zhang J, Fang L, Wang J, Fan W (2020). The prognostic value of COL3A1/FBN1/COL5A2/SPARC-mir-29a-3p-H19 associated ceRNA network in Gastric Cancer through bioinformatic exploration. J Cancer..

[22] Zeng XT, Liu XP, Liu TZ, Wang XH (2018). The clinical significance of COL5A2 in patients with bladder cancer: A retrospective analysis of bladder cancer gene expression data. Medicine (Baltimore)..

[23] Fischer H, Stenling R, Rubio C, Lindblom A (2001). Colorectal carcinogenesis is associated with stromal expression of COL11A1 and COL5A2. Carcinogenesis..

[24] Wang H, Chen H, Jiang Z, Lin Y, Wang X, Xiang J, Peng J (2019). Integrin subunit alpha V promotes growth, migration, and invasion of gastric cancer cells. Pathol Res Pract..

[25] Wu PL, He YF, Yao HH, Hu B (2018). Martrilin-3 (MA TN3) Overexpression in Gastric Adenocarcinoma and its Prognostic Significance. Med Sci Monit.

[26] Zhang C, Liang Y, Ma MH, Wu KZ, Dai DQ (2019). KRT15, INHBA, MA TN3, and AGT are aberrantly methylated and differentially expressed in gastric cancer and associated with prognosis. Pathol Res Pract..

[27] Kiyozumi Y, Iwatsuki M, Kurashige J, Ogata Y, Yamashita K, Koga Y (2018). PLOD2 as a potential regulator of peritoneal dissemination in gastric cancer. Int J Cancer..

[28] Kikuchi Y, Kunita A, Iwata C, Komura D, Nishiyama T, Shimazu K, Takeshita K, Shibahara J, Kii I, Morishita Y, Yashiro M, Hirakawa K, Miyazono K, Kudo A, Fukayama M, Kashima TG (2014). The niche component periostin is produced by cancer-associated fibroblasts, supporting growth of gastric cancer through ERK activation. Am J Pathol..

[29] Zhong H, Li X, Zhang J, Wu X (2019). Overexpression of periostin is positively associated with gastric cancer metastasis through promoting tumor metastasis and invasion. J Cell Biochem..

[30] Gugnoni M, Sancisi V, Gandolfi G, Manzotti G, Ragazzi M, Giordano D, Tamagnini I, Tigano M, Frasoldati A, Piana S, Ciarrocchi A (2017). Cadherin-6 promotes EMT and cancer metastasis by restraining autophagy. Oncogene..

[31] Sancisi V, Gandolfi G, Ragazzi M, Nicoli D, Tamagnini I, Piana S, Ciarrocchi A (2013). Cadherin 6 is a new RUNX2 target in TGF-beta signalling pathway. PLoS One..

[32] Li L, Huang S, Yao Y, Chen J, Li J, Xiang X, Deng J, Xiong J (2020). Follistatin-like 1 (FSTL1) is a prognostic biomarker and correlated with immune cell infiltration in gastric cancer. World J Surg Oncol..

[33] Li J, Wang W, Sun Y, Zhu Y (2020). CTLA-4 polymorphisms and predisposition to digestive system malignancies: a meta-analysis of 31 published studies. World J Surg Oncol..

[34] Zhang H, Li L, Yuan C, Wang C, Gao T, Zheng Z (2020). MiR-489 inhibited the development of gastric cancer via regulating HDAC7 and PI3K/AKT pathway. World J Surg Oncol..

[35] Cao F, Hu Y, Chen Z, Han W, Lu W, Xu J, Ding H, Shen X (2021). Circulating long noncoding RNAs as potential biomarkers for stomach cancer: a systematic review and meta-analysis. World J Surg Oncol..

[36] Yin XY, Pang T, Liu Y, Cui HT, Luo TH, Lu ZM, Xue XC, Fang GE (2020). Development and validation of a nomogram for preoperative prediction of lymph node metastasis in early gastric cancer. World J Surg Oncol..

